# Cost-effectiveness of Procalcitonin (PCT) guidance for antibiotics management of adult sepsis patients in the Egyptian context

**DOI:** 10.1186/s12913-024-11675-9

**Published:** 2024-10-17

**Authors:** Mohamed Metwally Mosly, Hesham Metwalli Mousli, Iman Mohamed Momtaz Ahmed, Mohamed Ibrahim Affify Abdou

**Affiliations:** 1 Department of Pharmacy Practice, College of Pharmacy, Arab Academy for Science, Technology & Maritime Transport (AASTMT), Alexandria, Egypt; 2 Department of Biomedical informatics and medical statistics – Medical Research Institute (MRI), Alexandria University - Alexandria, Alexandria, Egypt; 3 HOD Clinical Pharmacy at Andalusia Shalalt hospital, Alexandria, Egypt; 4 Critical Care Medicine - Cairo University, Cairo, Egypt

**Keywords:** Cost-Effectiveness, Procalcitonin, Guidance, Sepsis, Egyptian

## Abstract

**Background:**

Sepsis, which is described as a life-threatening organ malfunction brought on by an unbalanced host response to infection, continues to be a significant healthcare issue that affects millions of individuals each year. It is well-known that sepsis can affect anyone around the world, but the employed survey results showed that there are significant regional variations in sepsis incidence as well as mortality rates. Although there are no definite estimates for Egypt, the highest rates were in Low-Middle-Income Countries (LMICs).

Procalcitonin (PCT) is a host response marker with high specificity for bacterial infections, unlike C-reactive protein (CRP) or white blood cell count (WBC), which represent the traditional methods of detecting inflammation and infection. Its dynamic profile and superior prognostic prediction make it invaluable for assessing response to antibiotic treatment and improving clinical care for sepsis patients.

Our main purpose was to evaluate the cost-effectiveness of PCT guidance compared to no PCT guidance in the antibiotic management of adult sepsis patients according to the Egyptian context.

***Methods*:**

We developed a decision tree model to compare the PCT-guided antibiotic management duration endpoint versus the conventional laboratory culture-based antibiotic management in adult sepsis patients. We employed the“Delphi technique” to reach a satisfactory consensus regarding the resources attributed to each compared alternative. The primary measure of the study was the additional cost associated with each Quality-Adjusted Life Year (QALY) gained by sepsis survivors over a one-year time horizon.

Base-case, deterministic and probabilistic sensitivity analyses were conducted using TreeAge, Software.

**Results:**

Base-case analysis showed no dominance for either alternative and resulted in an Incremental Cost-Effectiveness Ratio (ICER) value of 297,783.57 Egyptian Pounds per Quality Adjusted Life Year (L.E/QALY) in favor of the PCT guidance alternative, Deterministic sensitivity analysis revealed that the highest impact magnitudes on ICER reside with seven input parameters, the top two parameters that had the most significant influence were the costs of ICU stay with and without PCT guidance. The CEAC showed a slightly higher probability in terms of acceptability in favor of the no PCT guidance choice along the WTP scale till reaching equal probabilities at the willingness-to-pay (WTP) value point of 390,000 (state currency) after which the - probability supports the PCT guidance choice.

**Conclusions:**

In the Egyptian context, PCT guidance has no cost-effectiveness domination over no PCT guidance in Antibiotics management for adult sepsis patients. This may be attributed to the high cost of PCT investigation that shall be resolved by standardization of its cost when applying the approach of DRG cost packages.

## Background

Sepsis is a serious condition that occurs when the body’s response to an infection that damages its own tissues and organs. It can be triggered by various infections, such as those affecting the lungs, urinary tract, skin, or abdomen. Although sepsis can affect anyone, certain groups including infants, the elderly population, people with chronic diseases, and those with weakened immune systems, are at higher risk. Sepsis continues to be a significant healthcare issue that affects millions of individuals each year. The condition can quickly escalate, leading to septic shock, organ failure, and death if not treated promptly. This underscores the critical need for effective management strategies, like procalcitonin (PCT) guidance, to improve patient outcomes [1]. 

As declared by the World Health Organization’s (WHO) Global Sepsis Epidemic and Burden of Disease report published in 2020, about 49 million cases of sepsis were diagnosed globally in 2017, and 11 million deaths were associated with sepsis, accounting for about 20% of global deaths [2]. It is well-known that sepsis can affect anyone around the world, but the employed survey results showed that there are significant regional variations in sepsis incidence as well as mortality rates [3]. Although there are no definite estimates for Egypt, one of the aforementioned WHO global studies concluded that the highest rates were in low-middle-income countries (LMICs). The study also concluded that in 2017, approximately 8.2 million deaths related to sepsis occurred in countries classified with low, low-middle, or middle Socio-Demographic Indexes (SDIs), representing 84.8% of the total deaths.

The average hospital-wide sepsis cost was estimated at over US$ 32,000 per patient, although these estimates were based almost exclusively on High-Income Countries (HICs) data [4].

Early diagnosis and appropriate initial treatment, including antibiotic therapy initiation and fluid replacement therapy, can remarkably improve sepsis prognosis. Additionally, proper patient monitoring during treatment, both for early treatment escalation in case of treatment failure and for treatment de-escalation in case of successful treatment response, has a significant positive effect on patient recovery [5, 6]. Since the clinical signs for sepsis monitoring are relatively ambiguous and can vary from patient to patient, the need for additional biomarkers that reflect specific physio-pathologic pathways evolved [7]. Serum PCT, such biomarker, was identified in the context of sepsis monitoring as a biomarker that can provide prognostic information for patients with sepsis, hence improving management. It was demonstrated through several studies that serum PCT levels rise in response to sepsis conditions and go down during recovery [8, 9]. Being a good reflection of the body’s response to sepsis, serum PCT became an excellent adjunctive aid in addition to conventional clinical and diagnostic investigations. However, the uptake of PCT guidance in Egypt remains limited, partly due to economic constraints and the preference of some traditional methods such as CRP and WBC counts. This limited adoption highlights the need for a thorough economic evaluation to determine the cost-effectiveness of implementing PCT guidance more broadly in the management of sepsis, especially considering the potential benefits in patient outcomes and healthcare resource utilization [10]. 

According to this approach, clinical care for patients experiencing sepsis can be significantly improved by using a specific host response marker that correlates specifically with the likelihood of bacterial infection. Among these markers, Procalcitonin (PCT) stands out due to its notable specificity for bacterial infections compared to other markers like C-reactive protein (CRP) or white blood cell count (WBC). Moreover, PCT demonstrates superior prognostic prediction capabilities and a dynamic profile that effectively tracks infections, thus proving valuable in assessing the response to antibiotic treatment [8, 11–15]. 

It was proven that the use of PCT-guided antibiotics regimen adjustment in Intensive Care Unit (ICU) patients suffering from sepsis is a safe and cost-effective option at an acceptable Willingness-to-Pay (WTP) threshold. While the cost-effectiveness of PCT guidance was demonstrated in high-income countries, such as the Netherlands and the U.S., It is crucial to evaluate its impact in different settings, including low- and middle-income countries, specifically in Egypt. Studies like those by Kip et al., and Collins et al. showed positive outcomes in their respective contexts, highlighting the need for similar research in other regions to better understand the economic implications and potential benefits of PCT guidance in sepsis management. Further investigation is necessary to assess how these findings can be applied to different healthcare environments and to inform cost-effective practices in diverse settings [16, 17]. 

## Methods

### Design and model

We developed a decision tree model to compare two approaches to antibiotic management in adult sepsis patients: one guided by PCT levels and the other based on conventional lab. culture results. This model allowed us to evaluate the potential differences in outcomes and cost-effectiveness between using PCT guidance and traditional culture-based methods for determining antibiotic regimen duration.

In the current study, outcomes were defined as non-recursive events within a limited time horizon. Four scores were used to obtain the final average EuroQol-5D (EQ-5D) values for the outcomes. These scores include: one-year mortality rates from the Stop Antibiotics on Guidance of Procalcitonin Study (SAPS), and Short-Form 36 (SF-36) scores obtained at ICU discharge, hospital discharge, and 3 and 6 months post-ICU discharge. This choice of modeling was based on its demonstrated effectiveness in evaluating discrete events with clear time limitations, supported by relevant literature sources. The decision tree approach provides a straightforward and practical method for analyzing such outcomes, aligning with the specific design parameters of our research. This methodological decision is supported by the literature, particularly the study conducted by Barton, Bryan, and Robinson (2004), which illustrates the selection of appropriate modeling techniques for the economic evaluation of healthcare interventions. This study underscores the suitability of the decision tree model in scenarios where the time horizon is explicitly defined [18, 19]. 

**Fig. 1:**
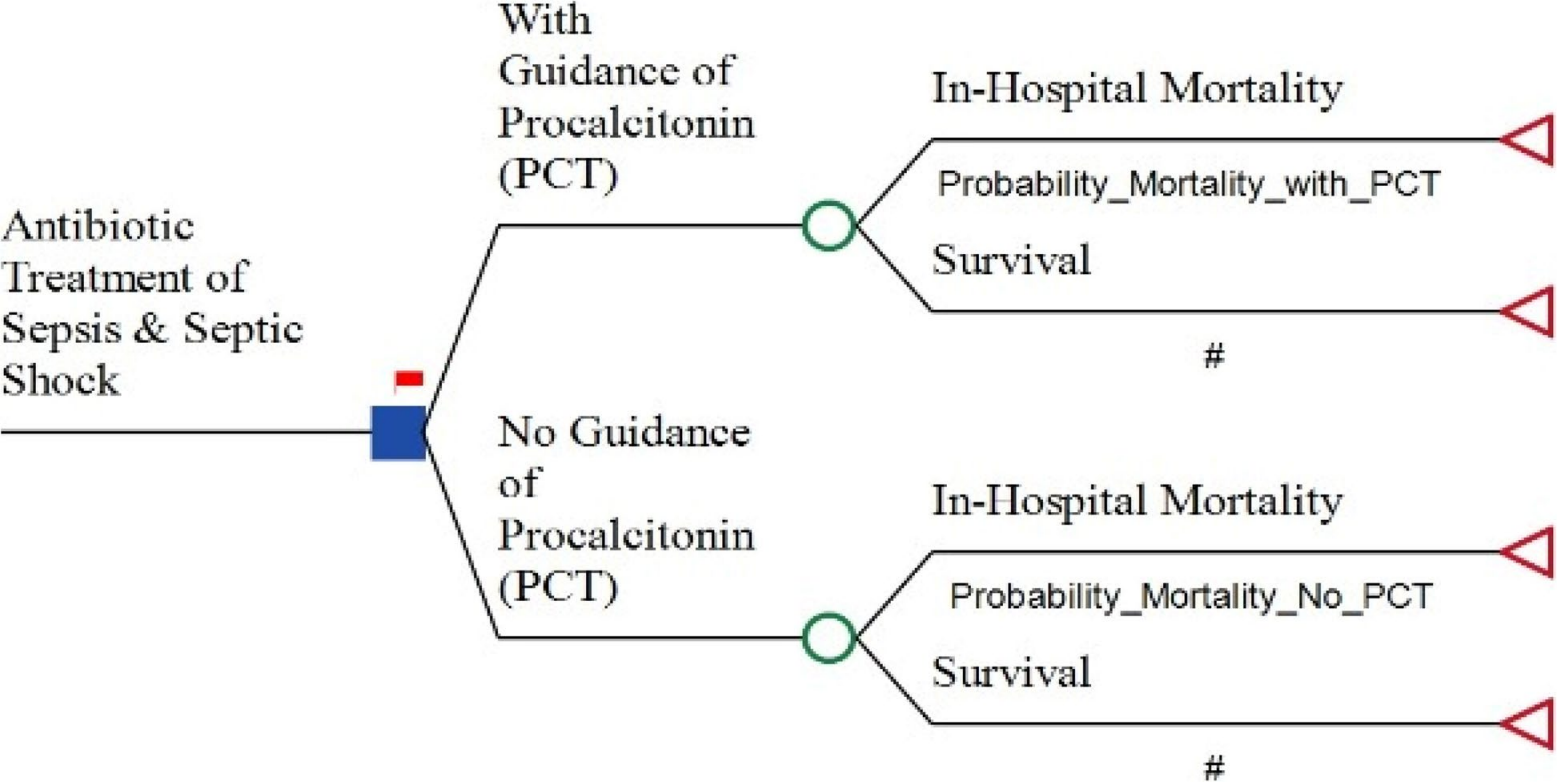
Decision tree model for evaluating cost effectiveness of Procalcitonin (PCT) guidance vs. No PCT guidance in Antibiotic treatment of adult sepsis patients

The decision tree model comparison arms encompass the cost data of either option as well as the effectiveness data, including probabilities, which were distributed to the relevant forms of decision nodes, chance nodes, and terminal nodes in a systematic approach to ease the comparison. The employed decision tree is shown in Fig. 1.

### Model inputs

#### Costs data

We estimated the cost data covering only the direct medical costs incurred during the hospital stay of adult sepsis patients. Researchers expended intensive efforts in collecting cost data associated with each compared alternative, reflecting the real-world situation in the Egyptian market. A paramount challenge researchers faced during the study was the variation in hospital stay duration and consequently costs, either associated with ICU or ward stays, as well as the lack of nationally standardized cost data for such services Additionally, the lack of nationally standardized cost data, such as standardized Diagnosis Related Group (DRG) cost packages in Egypt, could significantly impact the accuracy of the cost data. …therefore,

We employed the “Delphi technique” to reach a satisfactory consensus regarding the resources attributed to each compared alternative, reflecting the real patient sepsis management utilized resources to the maximum attainable extent. The Delphi technique is a structured method aimed at achieving a reliable consensus of opinion from a group of experts regarding a significant issue. It involves multiple stages of surveying, typically through intensive questionnaires, coupled with controlled feedback loops. Through iterative rounds of inquiry and feedback, the Delphi technique seeks to distill the collective wisdom of experts into a cohesive and reliable consensus, even when initial opinions may diverge [20]. 

In line with our cautious approach to using real-world data, we focused on accurately gathering the input data for the decision tree model. To do this, we employed the Delphi technique to survey a panel of experts, comprising twenty ICU consultants; ten from public sector tertiary hospitals, and ten from private sector institutions.

The panel of experts was asked, in terms of a two-round survey, to make the estimated cost data more reliable and to mitigate any potential biases through the integration of diverse perspectives of the involved field experts. This approach guaranteed the avoidance of any other factors affecting such as the number of proposed PCT tests conducted or PCT protocol adherence. Items that had not achieved consensus during the first Delphi round were included for a second round to develop the final list representing the final consensus and hence included in the analyses. A reliability measurement testing was conducted to test for the level of agreement between the expert panels included in the questionnaire process regarding their followed algorithm in adult sepsis patient’s routine management, either using PCT or without PCT utilization.

The reliability analysis included Cohen’s Kappa test, followed by Intraclass correlation test for items listed and the number of units utilized for each item, respectively. For both compared alternatives, the utilized resources were reported for a timeframe equivalent to the sepsis patient management total hospital stay, including both ICU and ward stays [21–23]. 

Table 1 presents the utilized resources that gained final consensus for each compared alternative. Following the development of item lists, comprehensive item cost data collection was conducted across various tertiary hospitals with adult sepsis management protocols within their scope of service. This approach aimed to ensure maximum market representativeness for the cost of each item in the management alternatives. Cost data were obtained from a panel of experts who participated in Delphi technique rounds. Complete cost data for the final item lists were collected from twenty different hospitals: ten public sector and ten private sector hospitals. This was done to ensure a comprehensive assessment of market costs. Intraclass correlation testing was then performed to evaluate the reliability of the collected cost data [24, 25], revealing a more than 90% agreement score on both single measures and average total measures levels.

**Table 1:** The cost-effectiveness decision tree model input parameters summary

Input parameters	Mean value	Minimum	Maximum
**A. Cost input parameters (L.E):**			
Total cost of ICU stay (No PCT guidance)	240,000	80,000	400,000
Total cost of ICU stay (With PCT guidance)	280,000	140,000	420,000
Total cost of Mechanical Ventillation (MV) (No PCT guidance)	11,100	7200	15,000
Total cost of Mechanical Ventillation (MV) (With PCT guidance)	9250	6000	12,500
Total cost of antibiotics regimen (No PCT guidance)	49,640	20,570	69,020
Total cost of antibiotics regimen (With PCT guidance)	35,040	14,520	48,720
Total cost of Hemodialysis (HD) (No PCT guidance)	21,000	4050	36,000
Total cost of Hemodialysis (HD) (With PCT guidance)	14,000	3700	24,000
Total cost of anaerobic culture	325	191	460
Total cost of blood cuture	790	408	1170
Toal cost of sputum culture	215	95	335
Total cost of urine culture	215	95	335
Total cost of Lab. PCT investigation	10,290	5320	15,260
Total cost of ward stay (No PCT guidance)	84,000	28,000	140,000
Total cost of ward stay (With PCT guidance)	80,000	40,000	120,000
**B. Incident probabilities:**			
Probability of In-hospital mortality (No PCT guidance)	0.298	0.235	0.364
Probability of In-hospital mortality (With PCT guidance)	0.218	0.171	0.264
**C. Outcome input parameters (QALY):**			
One year utility score (No PCT guidance)	0.47	0.43	0.51
One year utility score (With PCT guidance)	0.52	0.49	0.54

A. Cost data were collected using Delphi approach from twenty panelists of ICU consultants

B. Incident probabilities were obtained through extensive systematic literature review

C. Outcome input parameters (QALY) were obtained through extensive systematic literature review

Table 1 displays the mean, minimum, and maximum estimates for each listed utilized item in the sepsis management arms. All cost data are reported in terms of the 2023 Egyptian Pound currency (L.E).

#### Outcome data

The primary OUTCOME measure we focused on in our study was the Quality-Adjusted Life Year (QALY) gained by sepsis survivors. To define these utilities, we made use of data from a Dutch study [17] that combined one-year mortality rates from the Stop Antibiotics on guidance of Procalcitonin Study (SAPS) [26] together with a previous similar Dutch follow-up study [27]. We then converted the Short-Form 36 (SF-36) scores obtained at ICU discharge, hospital discharge, and 3- and 6-months post-ICU discharge into average EuroQol-5D (EQ-5D) values. By extrapolating these four utility scores and fitting an exponential function to the data, we estimated the utility one year after ICU discharge. The values for utility scores, together with their lower and upper limits, are shown in Table 1.

## Statistical analysis

### The economic evaluations were conducted using TreeAge, Software, LLC (Healthcare ProVersion 2023 R2.0)

#### Base-case scenario

The analysis adopted the payer perspective, focusing solely on direct medical costs incurred during the hospital stay. All other costs and a one-year post-discharge period were controlled for within the decision tree analytic model. Figure 1 illustrates the decision tree analytic model for the base-case estimates.

To assess cost-effectiveness, the study calculated the Incremental Cost-Effectiveness Ratio (ICER). This ratio measures the incremental cost of using PCT guidance per incremental Quality-Adjusted Life Year (QALY) gained, compared to not using PCT guidance. It provides an indicative measure of the additional cost associated with achieving one more QALY through the use of PCT guidance relative to not using the PCT guidance.

### Sensitivity analyses

#### Deterministic sensitivity analysis

**Fig. 2:**
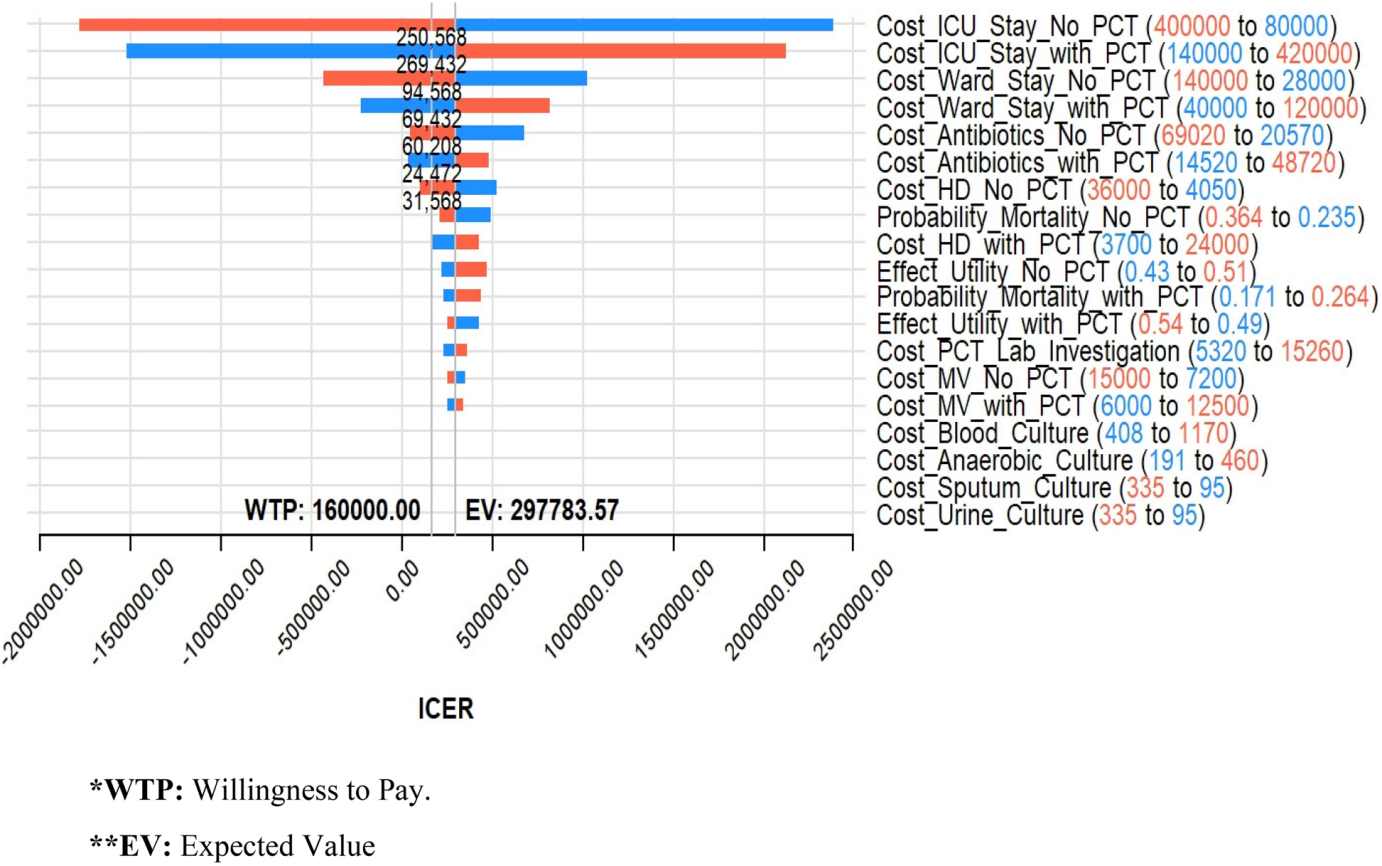
ICER Tornado diagram for input parameters deterministic sensitivity analysis. *WTP: Willingness to Pay. **EV: Expected Value

To test the effect of each parameter on the base-case ICER estimate, deterministic sensitivity analysis was conducted. Researchers allowed each of the nineteen input parameters, as dictated by the decision nodes in the decision tree, to vary individually within their defined ranges while controlling for other input parameters. Table 1 presents the nineteen parameters along with their respective range inputs. The outcomes of the deterministic sensitivity analysis were depicted using a tornado diagram, shown in Fig. 2.

#### Probabilistic sensitivity analysis

The study utilized a Monte Carlo simulation as a probabilistic sensitivity analysis (PSA) approach to evaluate the reliability of the base-case results by simultaneously varying all input parameters according to their relevant probability distributions. Cost data was assigned a Gamma distribution, while QALY scores were represented in terms of a Beta distribution. This probabilistic sensitivity analysis comprised one thousand iterations to derive the final study conclusions. The outcomes of the conducted PSA were presented in the form of a Cost-Effectiveness Acceptability Curve (CEAC), illustrating the likelihood of cost-effectiveness within a WTP range from 50,000 to 500,000 Egyptian Pounds (L.E). The specific WTP value for this study (160,000 L.E) was determined by researchers based on a conservatively accepted Gross Domestic Product (GDP) per capita value (1.5 times the local GDP per capita) for Egypt [28, 29]. 

## Results

### Base-case scenario

The base-case scenario comparison showed a total cost of 407,285 L.E. and a utility score of 0.32994 QALY for not utilizing PCT guidance, compared to a total cost of 430,125 L.E. and a utility score of 0.40664 QALY for utilizing PCT guidance. This indicates that PCT guidance was associated with higher costs but more QALYs compared to no PCT guidance, resulting in an ICER value of 297,783.57 L.E./QALY in favor of the PCT guidance alternative. The choice of utilizing the guidance of PCT showed lower Net Monetary Benefit (NMB) than choosing no PCT guidance (-365,062.6 and − 354,494.6 L.E. respectively) (Table 2).

**Table 2:** Base-case scenario results summary

Strategy	Cost	Incremental Cost	Effectiveness	Incremental Effectiveness	ICER	NMB
No Guidance of PCT	407,285		0.32994			-354494.6
With Guidance of PCT	430,125	22,840	0.40664	0.0767	297783.5724	-365062.6

### Deterministic sensitivity analysis

The deterministic sensitivity analysis identified seven input parameters with the highest impact on ICER among the nineteen parameters studied. These parameters include the cost of ICU stay with and without Procalcitonin (PCT) guidance, the cost of ward stay with and without PCT guidance, the cost of antibiotic regimen with and without PCT guidance, and the cost of Hemodialysis (HD) sessions without PCT guidance.

Conversely, the impact of input parameters related to the cost of various lab. culture types—such as blood culture, anaerobic culture, sputum culture, and urine culture—had no discernible effect on the estimated results. This lack of effect was observed because these costs appeared in both compared decision tree arms (Fig. 2).

### Probabilistic sensitivity analysis

**Fig. 3:**
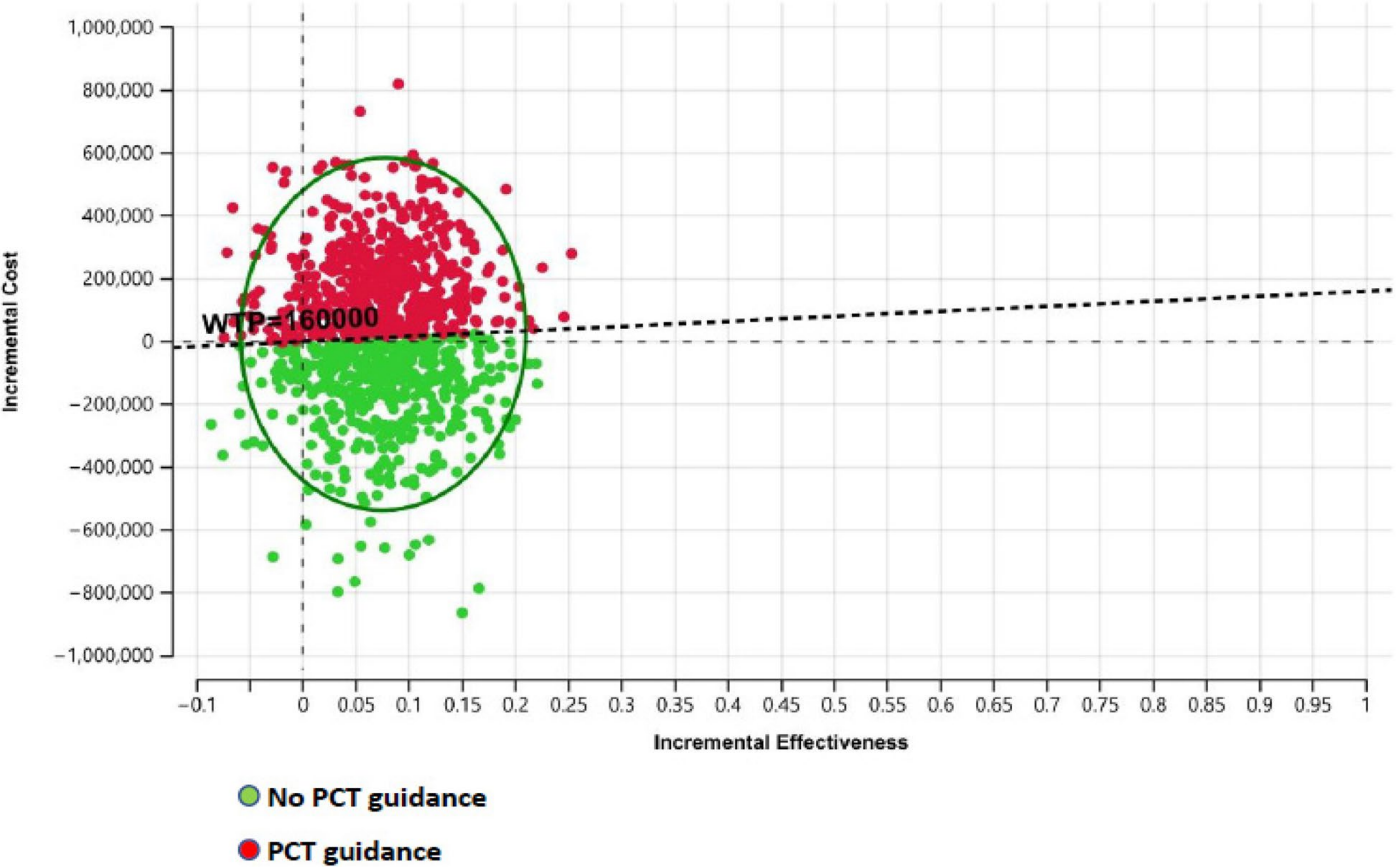
The Invremental Cost-Effectiveness (ICE) scatterplot of 1,000 iterations denoting probabilistic sensitivty analysis for Procalcitonin (PCT) guidance vs. No PCT guidance

The PSA outcomes include the Incremental Cost Effectiveness (ICE) scatterplot, which demonstrates the results of 1000 tested iterations, and the CEAC. In the ICE scatterplot, the no PCT guidance choice is represented by the green dots, while the PCT guidance choice is represented by the red-colored dots. The scatterplot indicates complete domination in favor of the no PCT guidance choice over the PCT guidance choice below the Egyptian WTP value (160,000 L.E.), as depicted in Fig. 3.

**Fig. 4:**
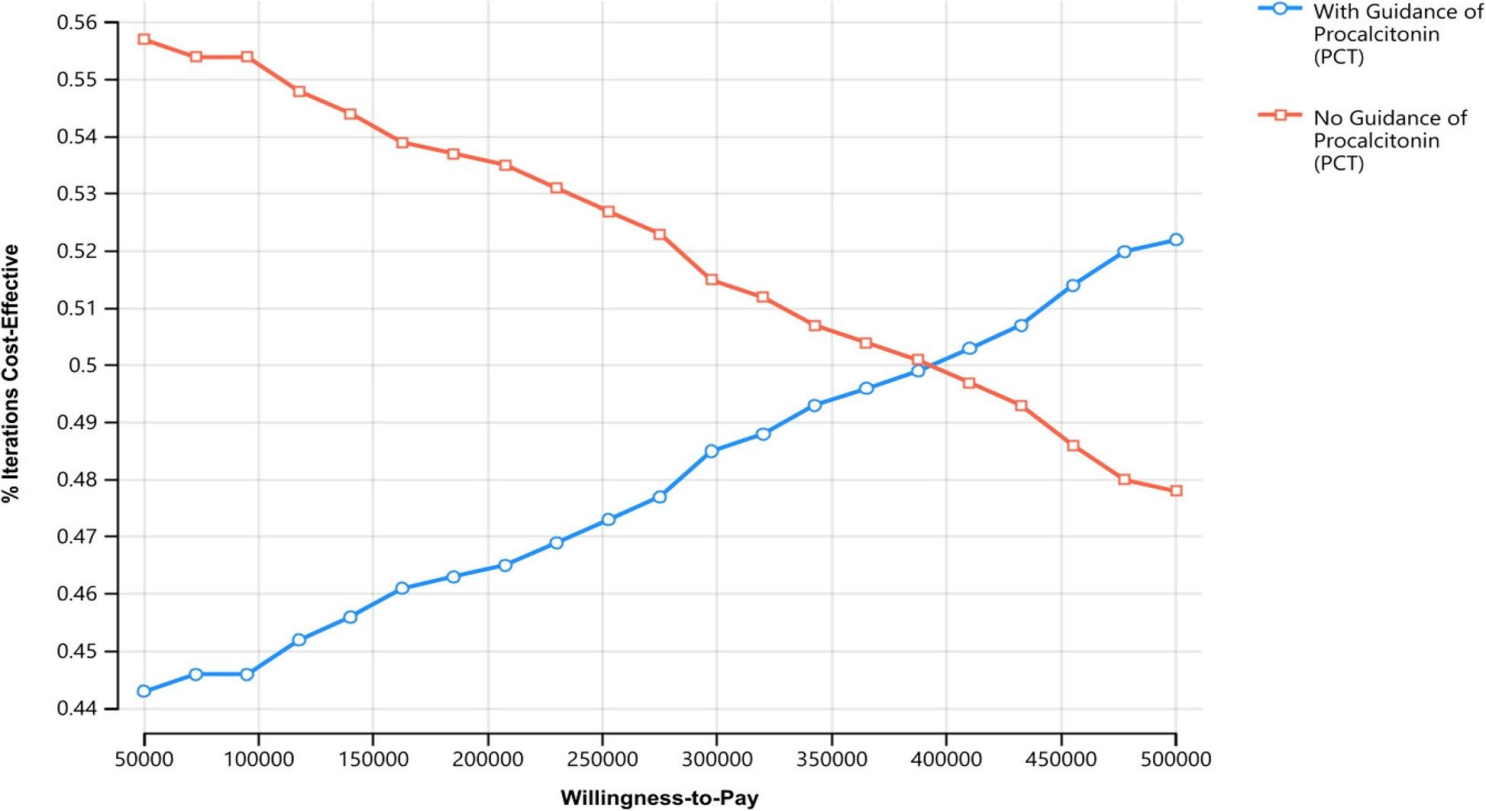
Cost-Effectiveness acceptability curve along the Willingness to pay (WTP) value scale

On the other hand, the CEAC illustrates a slightly higher probability of acceptability for the no PCT guidance choice along the WTP scale (approximately 56%) until reaching equal probabilities at the WTP value of 390,000. Beyond this point, the acceptance probability gradually increases in favor of the PCT guidance choice, reaching a maximum probability slightly higher than 52%, as shown in Fig. 4.

## Discussion

The results of the base-case scenario in our study indicated that PCT guidance led to higher costs but provided more QALYs compared to not using PCT guidance. It is assumed that the establishment of a universal health insurance system will comparatively lower the cost of procalcitonin (PCT) investigations, thereby shifting the probability of cost-effectiveness more towards following PCT guidance. Building on this assumption, the current ICER resulting from comparing PCT guidance versus no PCT guidance (297,783.57 L.E./QALY) is more likely to be accepted, considering the Egyptian Willingness-to-Pay (WTP) threshold (160,000 L.E.). This aligns sufficiently with the conclusion of the cost-effectiveness analysis conducted using real-world hospital data to evaluate the incorporation of PCT guidance into antibiotic management decisions for adult sepsis patients [30].

For the Egyptian healthcare sector, our study can be considered one of the few-if any-endeavors that aimed to utilize real-world cost data due to the lack of published or nationally available data sources. through the consensus-based Delphi technique. Reliability testing conducted supported the robustness of the Delphi technique outcomes. The expert panel participating in the Delphi rounds underwent a two-phase survey to achieve consensus on the recommended routine care for adult sepsis patients. The consensus obtained was pivotal in improving the reliability of the estimated cost data and reducing potential biases by integrating diverse perspectives from field experts. This approach ensured effective mitigation of other influential factors, such as the frequency of proposed PCT tests or adherence to the PCT protocol.

Given the urgent need for identifying the most efficient health interventions, particularly in middle- and low-income countries, to assist healthcare decision-makers, our study represents an ambitious effort to provide a practical decision-making support tool in accordance with recently published guidelines for such settings [31–34].

### Limitations

A potential limitation of our study may lie in depending on the literature-reported outcome data. This can be advocated by the immaturity of outcomes, especially utility scores evaluation studies in Egypt, and lacking a national value set for the interpretation of the health outcomes regarding sepsis patients, either with or without PCT approach utilization. So, it is justified for our study to utilize valid evidence for such outcome measurement depending on robust literature review [17, 36]. Another consequence of depending on literature outcome measures, regrettably, is that the long-term effect of PCT guidance regarding the survival and associated costs beyond the timeframe of one year couldn’t be addressed in our study. Moreover, due to the scarcity of survival and healthcare-associated costs incurred by sepsis survivors in general, extrapolation of the current study results may cause a high level of uncertainty in the findings [35].

## Conclusions

Our results indicate that PCT guidance (the alternative we evaluated) is less likely to be deemed cost-effective compared to no PCT guidance at the current WTP threshold in Egypt. However, the likelihood of PCT guidance being considered cost-effective increases as the WTP threshold rises to 390,000. It can be argued that the absence of standardized DRG cost packages in Egypt causes a significant variation in the PCT investigation cost consequently, the high cost of PCT-guided antibiotic regimen management for adult sepsis patients in Egypt significantly raises the ICER, thereby considerably decreasing the probability of this approach being the dominant option.

### Recommendations

It is recommended to conduct further research studies to address the aforementioned limitations. Additionally, proceeding with similar and more comprehensive economic evaluations is advisable. These evaluations can serve as evidence-based decision-making aids in Middle East and North Africa (MENA) region countries, enabling them to navigate and adapt to current economic fluctuations and turmoil for more efficient and effective healthcare choices. This approach aligns with the current international global direction [36]. 

## Data Availability

Not Applicable.

## Abbreviations

PCT: Procalcitonin
Lab.: Laboratory
LMICs: Low-Middle-Income Countries
SDIs: Socio-Demographic Indexes
QALY: Quality-Adjusted Life Year
ICER: Incremental Cost-Effectiveness Ratio
WHO: World Health Organization
HICs: High-Income Countries
WTP: Willingness to Pay
L.E: Egyptian Pound
SAPS: Stop Antibiotics on guidance of Procalcitonin Study
SF-36: Short-Form 36
EQ-5D: EuroQol-5D
CEAC: Cost-Effectiveness Acceptability Curve
NMB: Net Monetary Benefit
GDP: Gross Domestic Product
HD: Hemodialysis
ICE: Incremental Cost Effectiveness
DRG: Diagnosis Related Group
MENA: Middle East and North Africa
